# Interstitial Fluid in Lipedema and Control Skin

**DOI:** 10.1089/whr.2020.0086

**Published:** 2020-10-14

**Authors:** Marisol Allen, Michael Schwartz, Karen L. Herbst

**Affiliations:** ^1^ Department of Medicine, TREAT Program, University of Arizona, Tucson, Arizona, USA.; ^2^ Pasadena Plastic Surgery, Pasadena, California, USA.; ^3^ Herbst Clinic, Tucson, Arizona, USA.; ^4^ Limitless Therapeutics and Karen L. Herbst MD, PC, Los Angeles, California, USA.

**Keywords:** lipedema, leaky lymphatics, interstitium, glycosaminoglycan, extracellular matrix, lymphedema, microangiopathy

## Abstract

***Background:*** Fluid in lymphedema tissue appears histologically as spaces around vessels and between dermal skin fibers. Lipedema is a painful disease of excess loose connective tissue (fat) in limbs, almost exclusively of women, that worsens by stage, increasing lymphedema risk. Many women with lipedema have hypermobile joints suggesting a connective tissue disorder that may affect vessel structure and compliance of tissue resulting in excess fluid entering the interstitial space. It is unclear if excess fluid is present in lipedema tissue. The purpose of this study is to determine if fluid accumulates around vessels and between skin fibers in the thigh tissue of women with lipedema.

***Methods:*** Skin biopsies from the thigh and abdomen from 30 controls and 80 women with lipedema were evaluated for dermal spaces and abnormal vessel phenotype (AVP): (1) rounded endothelial cells; (2) perivascular spaces; and (3) perivascular immune cell infiltrate. Women matched for body mass index (BMI) and age were considered controls if they did not have lipedema on clinical examination. Data were analyzed by analysis of variance (ANOVA) or unpaired *t*-tests using GraphPad Prism Software 7. *p* < 0.05 was considered significant.

***Results:*** Lipedema tissue mass increases beginning with Stage 1 up to Stage 3, with lipedema fat accumulating more on the limbs than the abdomen. AVP was higher in lipedema thigh (*p* = 0.003) but not abdomen skin compared with controls. AVP was higher in thigh skin of women with Stage 1 (*p* = 0.001) and Stage 2 (*p* = 0.03) but not Stage 3 lipedema versus controls. AVP also was greater in the thigh skin of women with lipedema without obesity versus lipedema with obesity (*p* < 0.0001). Dermal space was increased in lipedema thigh (*p* = 0.0003) but not abdomen versus controls. Dermal spaces were also increased in women with lipedema Stage 3 (*p* < 0.0001) and Stage 2 (*p* = 0.0007) compared with controls.

***Conclusion:*** Excess interstitial fluid in lipedema tissue may originate from dysfunctional blood vessels (microangiopathy). Increased compliance of connective tissue in higher stages of lipedema may allow fluid to disperse into the interstitial space, including between skin dermal fibers. Lipedema may be an early form of lymphedema. ClinicalTrials.gov: NCT02838277.

## Introduction

Lipedema is a painful disease of excess loose connective tissue (LCT: fat), almost exclusively in women, that resists loss by diet, exercise, or even bariatric surgery.^1,2^ The trunk is generally spared in lipedema resulting in a disproportionate accumulation of fat on the limbs. Lipedema begins or is exacerbated in periods of hormonal flux when weight increases (puberty, pregnancy, and menopause), with surgery such as hysterectomy/oophorectomy, and at other times of weight gain.

Lipedema is graded by stage: In Stage 1, the skin is smooth but the tissue under the skin has a pebble-like feel, which suggests fibrosis in the tissue. Lipedema Stage 2 is like Stage 1 except there is more lipedema tissue, the skin has dimpling due to fibrotic changes in the skin and underlying LCT, and nodules are larger. In Stage 3, there are large lobules of lipedema skin and tissue.

Lipedema tissue mass, BMI, and metabolic disease worsen by stage,^3^ and lymphedema is more common in Stage 3 compared with Stage 2 and Stage 1.^4^ Interestingly, women with lipedema can also have hypermobile joints, especially prominent in Stage 3.^5^ Hypermobile joints suggest a connective tissue disorder such as hypermobile Ehlers Danlos spectrum disorder,^6^ associated with an increase in compliance and/or loss of elasticity to the tissue. In fact, people with the Williams-Beuren syndrome (OMIM 194050), who lack 1.5 million base pairs at 7q11.23 encompassing at least 17 genes, including the tropoelastin gene (*ELN*), can also present with lipedema-like tissue.^7^

Connective tissue is integral to the structure of blood and lymphatic vessels, and therefore, genetic changes may affect vessel compliance resulting in an increase in permeability especially when under increased pressure. Al-Ghadban et al. showed that women with lipedema have increased numbers of microblood vessels in fat (angiogenesis) that are dilated and convoluted, suggesting a structural defect that could increase the risk for dysfunction and permeability.^8^ This publication also demonstrated that lipedema skin and fat had more blood than lymphatic vessels, suggesting a limited ability to remove excess fluid generated by higher microvessel permeability^8^ in agreement with other published data.^9^

Excess fluid generated by microangiopathic blood vessels that is not picked up by lymphatic vessels would fill the interstitial space found throughout the body,^10^ resulting in enlargement of these areas. Vessels with greater permeability and increased size of interstitial spaces have been found in lymphedema,^11^ and capillaries surrounded by fluid suggesting hyperpermeability have been found in LCT of women with lipedema.^12^ Excess interstitial fluid may also make fat grow in lipedema as in lymphedema^13,14^ as lymph fluid is directly derived from interstitial fluid, which bathes cellular layers.^15^

Fluid shifts from the trunk to the lower body on standing increasing pressure in lower body arterial vessels,^16^ which could stress the structure of compliant vessels in lipedema, increasing interstitial fluid and fat growth on the thigh compared with the trunk. The aim of this study was to determine if an abnormal vessel phenotype (AVP) was present more often in the thighs compared with the abdomen in women with lipedema, in support of this hypothesis, and if blood vessels would have more AVP in women with lipedema compared with women without lipedema (controls). Spacing between dermal collagen fibers was used as a measure of increased interstitial fluid in the tissue.

## Methods

### Participants

All women were consented in writing before enrollment under a study approved by the University of Arizona Human Research and Protection Program. Women without lipedema were matched by age and BMI to women with lipedema as a comparator control population. Matching was accomplished with MedCalc Statistical Software (MedCalc Software Ltd, Ostend, Belgium). Women were considered to have obesity if their BMI was ≥30 kg/m^2^.

### Biopsies

Skin 5 mm punch biopsies were collected into zinc formalin from January 2017 to October 2019 from the thigh and/or abdomen of women with and without lipedema. Biopsies were processed into paraffin blocks, and then, slices cut and stained with hematoxylin and eosin (H&E).

### Abnormal vessel phenotype

**FIG. 1.**
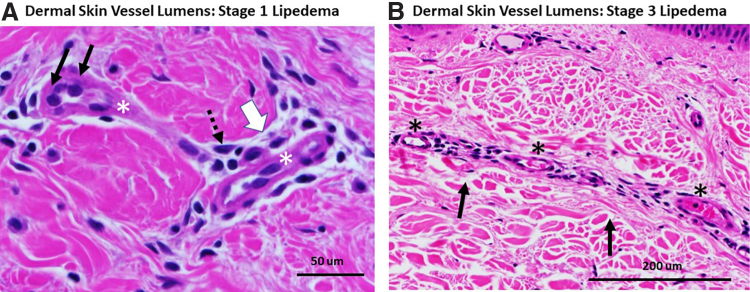
Dermal vessels from thigh skin of women with lipedema. **(A)** Lumens of two dermal vessels (*) from a woman with Stage 1 lipedema. Abnormal vessel phenotype: black arrows denote rounded endothelial cells. Dotted arrow points to perivascular immune cell infiltrate. The white arrow denotes the enlarged perivascular space. Note the close packing of the maroon-colored collagen fibers in the dermis with almost no space between. H&E. 60 × . **(B)** An elongated vessel with multiple lumens (*) in the dermis of a woman with Stage 3 lipedema; the base of the epidermis can be seen at the top of the picture. Note the lack of AVP and the wide spacing of collagen fibrils in the dermis (black arrows). H&E. 40 × to allow clear visualization of spaces. AVP, abnormal vessel phenotype; H&E, hematoxylin and eosin.

All vessels in the papillary and reticular dermis of skin samples were scored for abnormal or normal blood vessel phenotype by microscopy at 40 × magnification by two different observers. The number of abnormal vessels over the total number of vessels evaluated was multiplied by 100 to obtain a percent, and then, scores were averaged. A vessel was considered to have an abnormal phenotype if the following published criteria were met: (1) obvious perivascular space as in lymphedema^11,12^; (2) rounded endothelial cells^17,18^; and (3) perivascular immune cell infiltrate (Fig. 1).^8,19^

### Interstitial space

Five to eight images of the dermis at 40 × magnification were obtained avoiding empty spaces beyond the skin biopsy. Dermal collagen was highlighted and quantitated as percent field (density) using ImageJ,^20^ and then averaged and subtracted from 100 to get percent dermal collagen space. Space was used as a surrogate measure of the amount of interstitial fluid between collagen fibrils.

### Statistics

Data were analyzed by analysis of variance (ANOVA), linear regression, Pearson correlation, and unpaired Student's *t*-tests as noted in the Figures (GraphPad Prism Software 7, San Diego, CA). Data are presented as mean ± standard deviation. Significance was set at *p* < 0.05.

## Results

### Demographics

Women with Stage 1 lipedema were matched with controls by age and BMI; women with Stage 2 lipedema were also matched with controls, noted in Figures. Women with Stage 3 lipedema were included in the study and compared with controls but not all were matched to controls as they tended to be older and have a higher BMI than available controls, consistent with lipedema as a progressive disease (Table 1).

**Table 1.** Demographics of Women With and Without Lipedema

Demographics	Controls	Lipedema stages			All lipedema participants
		1	2	3	
Number (*n*)	30	19	40	21	80
Age (years)	38 ± 13	40 ± 9	49 ± 11^***^^,^^	51 ± 10^**^^,^^	47 ± 11
BMI (kg/m^2^)	28.3 ± 7	26.3 ± 3	33.4 ± 8^*^^,^^^	44 ± 8^***^^,^^^,+^	35 ± 9
White	24	19	35	19	63
Non-Hispanic	22	16	36	20	60

Versus controls: ^*^p = 0.01, ^**^p = 0.0005, ^***^p < 0.0001.

Versus L1: ^^^*p* = 0.01, ^^^^*p* = 0.002, ^^^^^*p* < 0.0001.

Versus L2: ^+^*p* < 0.0001.

BMI, body mass index; L1, lipedema Stage 1; L2, lipedema Stage 2.

### AVP in matched samples

**FIG. 2.**
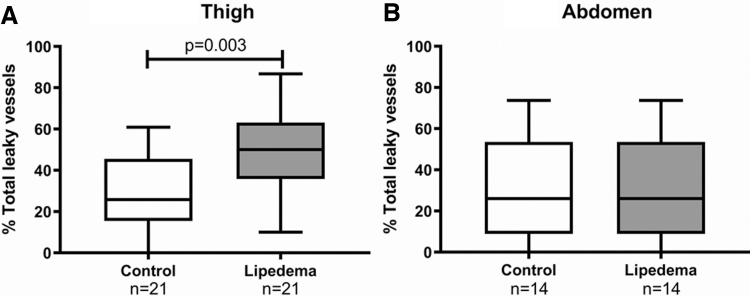
Leaky vessel phenotype in women with and without lipedema (controls) matched for age and BMI for the thigh **(A)** and matched separately for age and BMI for the abdomen **(B)**. Capped lines show significant differences between groups. Significance by unpaired *t*-test. BMI, body mass index.

When examining skin punch biopsy samples of abdomen or thigh skin from women with and without lipedema matched to controls by BMI and age, women with lipedema had significantly more AVP in the thigh versus controls, but similar numbers of AVP in the abdomen compared with controls (Fig. 2).

### AVP in thigh skin in all stages of lipedema versus controls

**FIG. 3.**
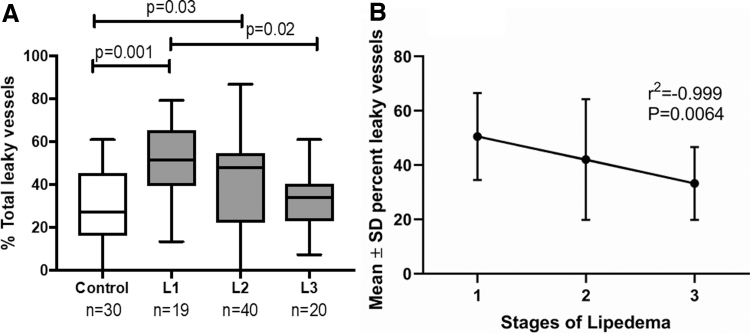
Women with Stage 1 lipedema have a greater percentage of AVP in the thigh than women with Stage 3 lipedema or controls. **(A)** Percent vessels with AVP in controls and all stages of lipedema by ANOVA. Capped lines show significant differences between groups. **(B)** Simple linear regression of means of percent abnormal vessels from all stages of lipedema. L1, lipedema Stage 1; L2, lipedema Stage 2; L3, lipedema Stage 3. ANOVA, analysis of variance.

As women with Stage 3 lipedema have more lipedema tissue than earlier stages, it was hypothesized that they would have more AVP than women with Stage 1 or 2 lipedema. However, there were significantly more AVP in thigh skin of women with Stage 1 or 2 but not Stage 3 lipedema versus controls (Fig. 3). Simple linear regression showed a strong negative correlation of AVP with stage (Fig. 3).

### AVP and obesity

**FIG. 4.**
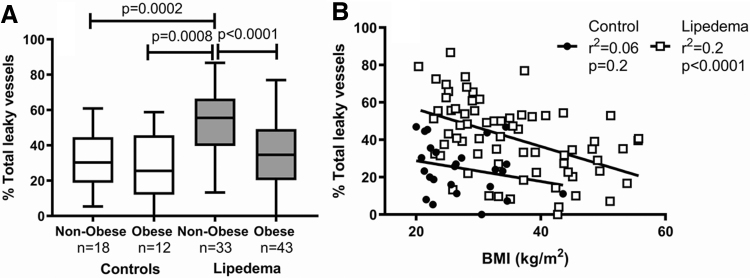
**(A)** Women with lipedema who are not obese have more vessels with a leaky vessel phenotype in the thigh compared with women with lipedema with obesity, who are obese or controls with and without obesity by ANOVA. Capped lines show significant differences between groups. **(B)** There was a negative relationship between BMI and leaky vessel phenotype in women with lipedema but not controls by Pearson correlation.

Women with lipedema without obesity had more AVP in thigh skin compared with women with lipedema and obesity, or controls (Fig. 4). The greater the BMI, the fewer AVP in women with lipedema (*r*^2^ = 0.23; *p* < 0.0001).

### Interstitial dermal spaces

**FIG. 5.**
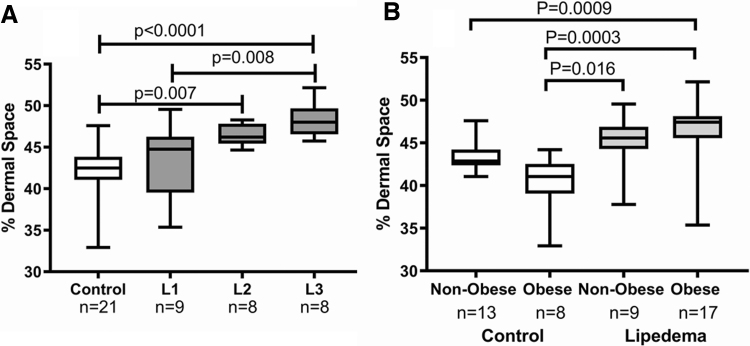
Space between collagen fibers in the dermis of the thigh in controls and women with lipedema with or without obesity. **(A)** Collagen space was significantly greater in women with Stage 3 lipedema compared with women with Stage 1 lipedema or controls, and women with Stage 2 lipedema compared with controls by ANOVA. **(B)** Dermal spaces were significantly larger in thigh skin of women with lipedema whether obese or not obese compared with controls. Capped lines show significant differences between groups. L1, lipedema Stage 1; L2, lipedema Stage 2; L3, lipedema Stage 3.

It was unclear why AVP were not as prominent in the thigh skin of women with Stage 3 lipedema. We hypothesized that increased compliance (change in interstitial fluid volume/change in interstitial pressure) in the tissue of women with Stage 3 lipedema allowed fluid to spread throughout the interstitial space. When examining abdomen and thigh dermal skin samples of women with and without lipedema, women with lipedema had significantly more space between collagen fibers in the dermis of the thigh (46% ± 3.5%) versus controls (42% ± 2.9%; *p* = 0.003). Dermal spaces were similar on the abdomen (42% ± 5.4%) compared with controls (43% ± 2.6%; *p* = 0.7). Women with Stages 2 and 3 lipedema had greater space between collagen fibers in thigh skin than controls (Fig. 5). There was no correlation between space between collagen fibers and BMI in women with lipedema (*r*^2^ = 0.03; *p* = 0.4) or controls (*r*^2^ = 0.15; *p* = 0.08).

## Discussion

Excess fluid that overwhelms lymphatic vessel function can result in lymphedema by the Starling rules for microvessels where lymphatic vessels supply the main exit route for fluid to leave tissue.^21^ Lipedema literally means fluid in the fat. In early stages, the fluid is nonpitting, so the fluid is dispersed in the tissue in the interstitial space and likely bound to glycosaminoglycans. Later, pitting edema can develop in lipedema similar to lymphedema. Blood vessel capillaries from lipedema tissue have been shown to be hyperpermeable,^12^ and thus could be one source of excess fluid entering the interstitial space. In support, there are increased blood vessels and microangiopathy in the skin and LCT of women with lipedema.^8^

We found AVP in the microvessels of the thigh but not abdomen of women with lipedema compared with controls. The thigh is an area of the body that has higher fluid column pressure when a person stands as fluid leaves the thorax.^16^ This pressure may be important in the development of lymphedema as disease onset is faster in the lower extremity after lymph node excision^22^ compared with the upper extremity.^23^ Excess lymph fluid makes fat grow,^13,14^ and therefore, it is not a stretch to consider that excess fluid in the tissue could also stimulate fat growth in lipedema.

The fact that so many women with lipedema have hypermobile joints suggests changes in connective tissue, including the structural connective tissue of vessels. Thus, higher hydrostatic pressure may pass more easily to the capillaries in the thighs of women with lipedema that then release excess fluid into the interstitial space. The abdomen as part of the trunk would not be as affected as the thigh due to lower fluid column pressure, consistent with our data of more AVP in the thigh versus the abdomen of women with lipedema.

Women with Stage 3 lipedema have more lipedema LCT. We hypothesized that women with Stage 3 lipedema would have a greater percentage of AVP in thigh skin vessels than women with Stage 1 lipedema. However, the opposite was found and in fact, the higher the stage, the lower the percent AVP in a highly statistically significant and linear manner. To better understand these data, we examined the interstitial space in the dermis. As in lymphedema,^24^ larger interstitial spaces were noted in the skin of women with Stage 3 compared with Stage 1 lipedema or controls. We hypothesize that dermal collagen may be more compliant in women with Stage 3 lipedema. Fluid that leaves vessels in the tissue of women with lipedema Stage 3 may enter the interstitial space and the poor elasticity (high compliance) of the tissue results in fluid stasis. This difference in compliance between Stage 1 versus Stage 3 tissue may reflect the contributions of different gene mutations. Mutations in genes that affect tissue compliance in the skin and vessels of women with Stage 3 lipedema may be different than mutations conferring compliance to microvessels of women with Stage 1 lipedema. For example, there are multiple reported genes that can confer a lipedema phenotype to tissue, and two of these genes affect tissue elasticity: *ELN* and *TNXB* (tenascin-X gene).^25^

The ability of dermal fibers to separate may be a marker of a hypermobile connective tissue disorder such as the hypermobile Ehlers Danlos hypermobile spectrum disorder. Our data also suggest that obesity alone does not drive the increased space between collagen in the dermis because women with lipedema with and without obesity had more space between collagen fibers in the dermis and greater AVP than controls with or without obesity. There are therefore inherent differences in the connective tissue between women with lipedema and controls, and obesity is not the driver for increased interstitial space or AVP.

Published data validate the findings of microangiopathy and angiogenesis in lipedema LCT. People with obesity (not lipedema) have lower adipose blood flow and calculated capillary diffusion capacity than leaner people,^26^ whereas in lipedema, vessel density is higher^8^ and vessels more permeable as shown by our work here. Lymphatic vessels were smaller and less numerous in published data on women with Stage 3 lipedema.^8^ This suggests that both microangiopathy and lymphatic dysfunction play a role in fluid accumulation, more so in later stages of lipedema than in earlier stages where a microangiopathy may play a larger role initially. A mouse model of obesity with fewer numbers of lymphatic vessels^27^ supports this model, suggesting that obesity is important in lymphatic failure in later stage lipedema.

Higher limb capillary pressures, tissue structure changes, more blood vessels with excess permeability and poor lymphatic outflow all likely contribute to fluid accumulation in lipedema.

The microangiopathy in lipedema microvessels may be caused by disturbance of the glycocalyx that lines all vessels and is composed of proteoglycans, glycoproteins, and associated glycosaminoglycans.^28^ The tissue of women with lipedema has higher sodium concentrations.^29^ High sodium disturbs the glycocalyx barrier function of endothelial cells and predisposes the endothelium to inflammation.^30^ Low-sodium diets may improve the glycocalyx.^31^ More research is needed in this area.

If a microangiopathy and interstitial fluid bound to glycosaminoglycans are present early in lipedema tissue, treatment should aim at supporting the vessels in the legs by the use of the following: compression garments; hands on therapy to move fluid, sodium, and other prelymph components out of the tissue into lymphatic vessels; flavonoids and other anti-inflammatories from food and supplements to help reduce inflammation of the microvessels^32^; and possibly a low-salt diet to decrease glycosaminoglycan content.^31^

The data in this article were based on an AVP and collagen spacing that if incorrect as markers of leaky vessels and increased interstitial fluid, respectively, would negate our findings. However, our phenotype was based on published data demonstrating perivascular spaces around capillaries in fat tissue in lipedema,^12^ perivascular spaces,^11^ and spaces between collagen fibers in the dermis^24^ in lymphedema, perivascular immune cell infiltrate in the skin in lipedema,^8,19^ and endothelial cell rounding as a marker of increased paracellular transport.^17,18^

## Conclusion

Microangiopathy is likely present in women with early-stage lipedema that manifests more often in tissues exposed to higher hydrostatic pressure such as the thighs. In later stages, lymphatic dysfunction becomes an important contributor to tissue fluid. This excess fluid can reside around vessels or among tissue fibers. The increased fluid would account for the heaviness of the tissue as is often found in the legs of women with lipedema. Lipedema may be in the spectrum of lymphedema. The interstitial space and its glycosaminoglycans, the endothelial glycocalyx, and structure of the lipedema tissue need to be studied further.

## Abbreviations Used

ANOVA: analysis of variance
AVP: abnormal vessel phenotype
BMI: body mass index
H&E: hematoxylin and eosin
LCT: loose connective tissue
